# Protocol of a scoping review on knowledge translation competencies

**DOI:** 10.1186/s13643-017-0481-z

**Published:** 2017-05-02

**Authors:** Anastasia A. Mallidou, Pat Atherton, Liza Chan, Noreen Frisch, Stephanie Glegg, Gayle Scarrow

**Affiliations:** 1 School of Nursing, University of Victoria, 3800 Finnerty (Ring) Road, Victoria, BC V8P 5C2 Canada; 2 School of Nursing, University of Victoria, PO Box 1700 STN CSC, Victoria, BC V8W 2Y2 Canada; 3 Alberta Innovates & University of Alberta, 1500 10104-103 Avenue, Edmonton, AB T5J 4A7 Canada; 4 Sunny Hill Health Centre for Children, 3644 Slocan Street, Vancouver, BC V5M 3E8 Canada; 5 Michael Smith Foundation for Health Research, 200 - 1285 West Broadway, Vancouver, BC V6H 3X8 Canada

**Keywords:** Knowledge translation, competencies, knowledge, skills, attitudes, scoping review, evidence-based practice

## Abstract

**Background:**

Knowledge translation (KT) activities can reduce the gap between “what is known” and “what is done”. Several factors hinder or facilitate KT activities including individual characteristics and organizational attributes; we will focus on individual healthcare professional modifiable characteristics. The purpose of this scoping review is to summarize knowledge on KT competencies for knowledge users, knowledge brokers, and knowledge producers/researchers to support evidence-based practice (EBP) and inform policy and research in health. Our objectives are to explore the relevant theoretical and empirical literature; map the publications for key themes and research gaps of KT competencies, and interventions for enhancing KT competencies; summarize and disseminate findings; produce an action plan and research agenda; and develop self-assessment tools (the KT Pathways) for professional development for our three target audiences.

**Methods:**

The scoping review method will guide our study by following six stages: formulating the research question; identifying relevant studies; selecting the literature; charting the data; collating, summarizing, and reporting the results; and developing a KT plan and consulting stakeholders involved in the fields of KT, EBP, evidence-informed policy-making, and/or research. We will include empirical and theoretical/conceptual peer-reviewed and grey literature in health that examine knowledge user, knowledge broker and knowledge producer KT competencies. Publications written in the English language and published after 2003 only will be considered. Our multidisciplinary research team will collaborate using technology (i.e., WebEx for discussions and a Web 2.0 website for storing documents). Our KT plan consists of an Advisory Group and dissemination plan of the findings.

**Discussion:**

We expect the identified KT competencies to contribute to the KT science by providing positive outcomes in practice, policy, education, and future research. Incorporation of the core KT competencies may enhance safety, effectiveness of clinical care, and quality of health outcomes; contribute to and facilitate collaboration among practitioners, knowledge users, knowledge brokers, researchers, employers, and educators; improve education of healthcare professionals and inform policy-making process; benefit practitioners by guiding their KT professional development to become effective at moving evidence into practice and policy; guide suitable interventions and strategies to enhance KT activities in the health sector; and direct future research.

**Electronic supplementary material:**

The online version of this article (doi:10.1186/s13643-017-0481-z) contains supplementary material, which is available to authorized users.

## Introduction/background

Resistance to scientific discovery and innovative ideas is a natural response during the critical process of both scientific discoveries and innovative ideas [2, 28] that results in enlarging the gap between research findings and practice and/or policy-making, since research findings are not easily and well accepted. Many landmark documents have illustrated the primary need to use knowledge in routine practices including *Crossing the Quality Chasm* [19] and *Health Professions Education: A Bridge to Quality* [17]. Also, influential organizations such as the *Agency for Healthcare Research & Quality* (AHRQ) and the World Health Organization (WHO) have identified the need for supports for evidenced-based practice and evidence-informed policy-making. The AHRQ (as of December 2014) has funded 13 Evidence-based Practice Centers (EPCs) to answer clinical research questions for improving quality of healthcare and patient outcomes (http://tinyurl.com/j4skdzl), while in 2005 the WHO established an evidence-informed policy network (EVIPNet) to promote the systematic use of health research findings in policy-making (http://www.who.int/evidence/about/en).

Knowledge translation (KT) process has the potential to reduce this gap between “what is known” and “what is done”. Although a paradigm shift toward valuing KT, evidence-based practice (EBP), and evidence-informed policy-making has improved the use of evidence in the last few years, variations in practice and policy still exist [26, 27]. Also, despite efforts to support KT and promote EBP and evidence-informed policy-making, using numerous KT frameworks [7, 33], knowledge translation activities are not yet systematically used worldwide.

A number of factors have been examined that hinder or facilitate KT activities including individual characteristics and organizational attributes. For practitioners, having a positive attitude toward research and possessing basic knowledge and skills related to KT activities is critical, yet inadequate for changing behavior [25]. In a systematic review, Estabrooks et al. [11] found that use of research findings in practice is associated with individual healthcare practitioner beliefs and attitudes toward research. Interventions to strengthen healthcare professionals’ attitudes and beliefs toward KT activities may trigger intention to systematically incorporate the best available evidence into routine clinical practice. For policy-makers, factors at the individual level that significantly predict research use in certain public health decision-making contexts include research skills and intention to use research findings in the near future (i.e., the next 12 months) [34]. For researchers, requirements by funding agencies to include KT activities as part of their research-funding applications, partly to demonstrate accountability for public dollars spent, have increased in recent years that facilitate EBP and evidence-informed policy-making [18, 30]. For knowledge brokers, personal attributes are important for KT activities and tasks [3]. People with a certain type of personality (e.g., flexible, curious, self-confident, imaginative, intuitive, inquisitive), analytical skills, and capability in managing human intellect can work well in facilitating KT activities [4]. Lomas [23] describes knowledge broker (KB) attributes and skills in more details including the ability to clearly communicate, understand organizational and work environment cultures, assess research findings in various formats, facilitate, mediate, and negotiate among stakeholders. In addition, the findings of a study on knowledge brokering in public health include personal characteristics that are central in a KB’s role to implement knowledge and change behaviors. Particularly, the authors describe KBs as “skilled in terms of appraising evidence”, encouraging people who work with them, inspiring respect and trust, supportive, approachable, and able to mitigate anxiety and contextualize research findings in various contexts [31]. Organizational factors including workplace culture, resources, and support are also fundamental for KT activities and EBP [9, 10, 12, 13].

Focusing on at the individual level of healthcare professional (modifiable) characteristics, we argue that competency-based education and other multifactorial interventions may trigger positive attitude toward research and enhance knowledge and skills. In general, focus on competency-based education for healthcare professionals is important and transparent that allows for innovative and student-centered learning and teaching processes, and facilitates new opportunities to emerge for redesigning health systems [14]. The importance of developing knowledge, skills, and attitudes (i.e., competencies) is also emphasized in the Lancet commission report “*Health Professionals for a New Century: Transforming Education to Strengthen Health Systems in an Interdependent World*” [14]. The connection between education and health systems and the need for transformative professional education are also highlighted to promote new approaches for optimizing health systems. Educational systems must produce well-equipped graduates, who will meet patient and population needs by sharing learning activities and strengthening research competencies to build upon the knowledge base about innovations and EBP and evidence-informed policy-making. Another positive contribution of competency-based education is its potential to transfer knowledge and skills learned at school into the needs of society [20].

As such, we are attempting to compile a set of core KT competencies [27] for knowledge users, knowledge brokers, and knowledge producers (researchers) to support EBP and evidence-informed policy in health (for a brief description of these terms please see Additional file 1). Incorporation of KT competencies into the education and health systems (e.g., job expectations, performance appraisals, promotion procedures) may positively influence KT activities and KT learning needs of these three groups of audience. As a result, we expect to note development of comprehensive training programs, implementation of research findings, consistency and quality of healthcare, and reduction of health system expenses [26]. The definitions of the primary concepts used in this protocol of the scoping review are analytically described in Additional file 1.

### Purpose and objectives

The purpose of the proposed scoping review is to summarize existing knowledge on KT competencies for three discrete audiences (i.e., knowledge users, knowledge brokers, and knowledge producers) in health to support EBP and inform policy and research. Our primary research question is “*What are the core KT competencies of knowledge users, knowledge brokers, and knowledge producers/researchers and the interventions and/or strategies to teach and reinforce those competencies?*” Our secondary research question is “*Is it possible these identified core KT competencies to include in self-assessment tools for professional development for each of the three target audiences to succeed in the KT field?*” Particularly, our main objectives are to:

- Systematically explore the extent of relevant theoretical and empirical literature (e.g., range, focus, nature of sources, volume) on KT competencies for knowledge users, knowledge brokers, and knowledge producers in health.
- Map the publications by identifying definitions (e.g., key themes) of knowledge users’, knowledge brokers’, and knowledge producers’ KT competencies; research gaps; and potential interventions for boosting KT competencies.
- Summarize and disseminate review findings to the three groups in relevant fields (e.g., health research, nursing, medicine, occupational therapy, health policy).
- Produce an action plan and a research agenda for three distinct areas: designing future primary studies on areas that are needed, conducting systematic reviews on topics that already adequate knowledge exists, and integrating KT activities and recommendations for policy about the use of the three groups’ KT competencies in the health sector.
- Develop self-assessment tools (the KT Pathways) for professional development for our three target audiences to succeed in the KT field. These tools will include core KT competencies identified through the current scoping review, along with relevant resources to support the development of the identified KT competencies.

## Methods

To address the purpose and objectives of the proposed study, we will use the scoping review method described by Arksey and O’Malley [1] and further developed by Levac et al. [21]. This method includes six stages: (a) formulating the research question; (b) identifying relevant studies; (c) selecting the literature (an iterative process); (d) charting the data; (e) collating, summarizing, and reporting the results; and (f) developing a KT plan and consulting interested stakeholders. We will also follow the PRISMA-P checklist [29]. As part of the integrated knowledge translation approach, we will further involve and consult knowledge users, knowledge brokers, policy-makers, and researchers involved in doing, facilitating, and/or producing KT, EBP, or evidence-informed policy-making and research throughout the research process to provide various perspectives, meaning, and applicability of the review findings. We have not registered this proposal with PROSPERO, because this study is a scoping review; not a systematic review that is usually registered with PROSPERO.

### Formulating the research question

The research question for this scoping review will be finalized in consultation with relevant stakeholders, who have already been involved in the initial steps of this review and contributed to the discussion and the proposal writing process. The research question and objectives have been shaped as it is stated in the previous section.

### Identifying relevant studies

We will further expand the targeted search strategy that was initially developed in consultation with a research librarian (member of our research team) and refine the parameters of our search strategy. For the purposes of this scoping review, we will systematically search the academic (peer-reviewed) and grey literature to identify relevant publications (for details, see Additional file 2). Using subject headings and keywords, our comprehensive and systematic search strategy will include searches in and of:

- Health, healthcare, and a variety of interdisciplinary electronic databases with this focus such as PubMED, Ovid MEDLINE, Cochrane Library, Scopus, EMBASE (Ovid), Cumulative Index to Nursing and Allied Health Literature (CINAHL) EBSCO, NEOS Library Consortium Catalogue, and Theses Canada.
- Grey literature sources such as existing networks (e.g., InspireNet), relevant organizations (e.g., Canadian Institutes of Health Research; Canadian Foundation for Healthcare Improvement—formerly Canadian Health Services Research Foundation), conferences, government, NGOs, health research websites (e.g., National Collaborating Centre for Methods and Tools, World Health Organization), and databases specific to grey literature (e.g., KT Clearinghouse, Evidence-Informed Health Care Renewal or EIHR portal).
- Hand-searching of relevant specialized key journals (e.g., Implementation Science).
- Reference lists in publications identified in (a), (b), and (c).
- Personal contacts of working group and stakeholder groups.

*Search terms* will include controlled vocabulary and various keywords and terms related to (1) KT keywords such as knowledge translation, knowledge utilization/use, research use; (2) KT competencies such as knowledge, skills, and attitudes related to knowledge translation in health or healthcare. Because of the lack of indexed subject headings, and a large amount of literature expected to be related but not on-topic, the search terms will primarily be used for the title and abstract search. Search limits will be applied in language (English only), publication date (between 2003 to present to focus the search in accordance with the relatively recent and prominent development of the KT field), and publication status (e.g., in review, accepted, in press). Search results will be imported into a bibliographic manager (i.e., Mendeley) and duplicates will be removed.

### Selecting the literature

In this iterative process, retrieved search results will be reviewed for inclusion or exclusion. Refining the search strategy might take place at the update of the literature search. At least two investigators will independently screen the titles and abstracts of all publications retrieved, based on pre-determined inclusion criteria. Publications identified as potentially relevant to this review will be retrieved in full text and reviewed against the same inclusion criteria. Disagreements regarding a publication inclusion will be resolved through discussion between the two reviewers or third party adjudication.

### Inclusion/exclusion criteria

#### Inclusion

All empirical and theoretical/conceptual peer-reviewed publications in health as well as documents from the grey literature that examine knowledge user, knowledge broker, and knowledge producer KT competencies will be considered for inclusion. Specifically, each publication has to:

- Be an empirical or theoretical/conceptual peer-reviewed publication in the health sector, or a document from the grey literature;
- Include both concepts and/or sub-concepts of *knowledge translation* or any other similar term (e.g., knowledge utilization, knowledge use, knowledge transfer) and *competency* (i.e., knowledge, skills, attitudes) or any component of competencies that refer to knowledge user, knowledge broker, or knowledge producer competencies in KT; and
- Have an abstract and purpose clearly stated (for empirical and conceptual publications only); grey literature will still be reviewed in the absence of an abstract.

If several publications are based on the same dataset, all relevant to KT competency publications will be included.

#### Exclusion

Publications written in non-English language and published before 2003 will not be included. Restrictions according to status of publication (e.g., in review, accepted, in press) will not be applied. Other relevant KT articles will be held in a separate folder to be reviewed as background documents to support the analysis of our knowledge synthesis study.

### Classifying the literature

We will classify the publications into empirical and theoretical peer-reviewed papers (including reports and reviews), and in grey literature using spreadsheets. We will develop a data extraction instrument for this study using standard formats (Additional file 3). Charting will be an iterative process at the beginning of the data extraction stage. Data will be entered into Microsoft Excel spreadsheets in tabular format. Prior to commencing the full data extraction, at least two investigators will independently extract data from a sample of publications (e.g., ten) to determine the consistency, accuracy, and completeness of their approach with the purpose of the review; and to refine the form for capturing all the details of quantitative and qualitative study designs. Data to be extracted include study design, theoretical framework, participant characteristics (demographics), KT competencies (i.e., knowledge, skills, and attitudes), study findings, and intervention/strategy details. Discrepancies in data extraction will be discussed and resolved by consensus.

### Synthesizing and summarizing findings

Theoretical and empirical literature will be summarized as a traditional integrative review [6, 24, 32]. We will identify commonalities in constructs across studies, map them, and collate the data extracted from empirical studies. Then, we will summarize publications and their characteristics in a table (e.g., frequency and type of publications, variables used and defined, study design, type of intervention, measured outcomes, use of theoretical framework) that will constitute our map of the literature. The grey literature will be also summarized in an integrative review. Then, we will combine the findings from both kinds of the literature (academic and grey) accordingly using narrative and descriptive summaries as well as an interpretive synthesis [8].

The KT competencies will be summarized from both the academic and grey literature in a holistic approach in terms of knowledge users, brokers, and producers/researchers; but they will be grouped separately in three categories according to the component of competencies (i.e., knowledge, skills, attitudes). The rationale for this approach is that there are limited number of primary studies that separately examine KT competencies for knowledge users, knowledge brokers, and knowledge producers/researchers. However, based on this knowledge synthesis, we will develop the KT Pathways (a self-assessment tool) for each stakeholder separately.

### Reporting results

We will report the findings of the review using tables describing the characteristics of each publication that will be included in our scoping review and classify them according to their characteristics (e.g., study design, intervention). Additional tables will classify the included publications according to their main characteristics such as participants, study setting, study design, study intervention (if applicable), theoretical frameworks used, KT competencies (i.e., knowledge, skills, attitudes) identified in the publications, and findings. We will also draw a conceptual diagram (mind mapping) that will include all identified core KT competencies, to illustrate the relationships among the components of the various publications. The mind mapping will be helpful in designing future systematic reviews focused on interventions that are intentionally used to hone KT competencies in all three audiences. Peer-reviewed (academic) publications and grey literature documents will be described and synthesized separately, but both will be used to identify the core KT competencies.

### Integrated knowledge translation plan

Our multidisciplinary research team consists of researchers, knowledge users, a knowledge broker, and a librarian. The research team will regularly collaborate using technology (i.e., WebEx). The discussions, decisions, and all relevant documents will be stored on a Web 2.0 website using infrastructure provided by InspireNet (INnovative health Services & Practice Informed by Research & Evaluation Network), a virtual research and knowledge network. Our scheduled KT plan consists of two parts:

1. Advisory Group—The “linkage and exchange model” [22] informed our establishment of an Advisory Group (Additional file 4) to ensure that a broad range of stakeholders in two Canadian provinces (Alberta and British Columbia) will contribute to the research process and deliverables of the study. The research team will engage regularly with the Advisory Group members via newsletters and technology to solicit their input on a regular basis. We expect our multidisciplinary Advisory Group members to provide feedback on the research process and deliverables of the scoping review; their expertise and/or insights they might have on the needs of knowledge users, KBs, and researchers as well as relevant KT competencies that are required for effective EBP and evidence-informed policy; and strategic advice on the interpretation of the study findings and appropriate dissemination ways to local, national, and international interested individuals and organizations.
2. Dissemination of the findings—Drawing from the Knowledge-to-Action (KTA) framework [15] and the steps of a Planned Action Model [16], we will integrate and use the Dissemination Planning Tool [5] to create a dissemination plan beyond the traditional academic methods. For example, opinion leaders can offer an innovative approach to sharing knowledge that has the potential for greater effectiveness than passive approaches (e.g., conference presentations). Our constructed dissemination plan will be used at the end of the scoping review, when the findings are known and the KT Pathways professional development tools are fully developed, and based on the needs and interests of our intended users/audiences. We will also use the Dissemination Planning Tool [5] with our Advisory Group members to “plant the seeds of interest” ([5], p. 85). Specifically, we plan to develop an interactive KT plan by (a) describing key messages emerging from this review; (b) determining the target audiences for each message; (c) identifying the best messenger for each message and audience; (d) involving the Advisory Group in the development of the dissemination plan and process of spreading the messages arising from the scoping review; and (e) using diverse approaches to disseminate the study findings.

## Discussion

The proposed knowledge synthesis study contributes to the literature with the potential to influence practice, education, policy, and future research. Incorporating the identified KT competencies into each individual healthcare professional practice could have several positive outcomes. First, the use of KT activities as a routine practice by healthcare professionals in various roles (e.g., knowledge brokers) can enhance the safety, quality, and effectiveness of clinical care. Second, clinical practice conducted using the identified KT competencies may eliminate variability of care across healthcare organizations that, in turn, will promote quality of health outcomes. Third, the findings of this review and use of identified KT competencies may result in greater involvement and will help to bring together and facilitate collaboration among healthcare practitioners, knowledge brokers, knowledge producers, employers and educators, and policy-makers. Fourth, core KT competencies have the potential to improve education of healthcare professionals by assisting educators in focusing training on these core KT competencies. Fifth, incorporation of the identified KT competencies in job expectations, performance appraisals, and promotion procedures is a direct and clear benefit for both healthcare outcomes and professionals. For example, knowledge brokers and researchers will have guides for KT professional development that will help them to be effective at moving evidence into practice and policy. Sixth, KT competencies can guide suitable interventions and strategies to enhance KT activities in the health sector. Finally, the identified research gaps among the study findings may provide guidance for future systematic reviews and research projects.

### Limitations

Although we will follow a rigorous and sound process and methods for this scoping review, we anticipate a number of limitations. First, a language limitation will be inevitable, because of the inclusion of publications written only in the English language. Second, our broad search strategy might be associated with a lower precision on the purpose of the review that may result in a large number of redundant references. Third, using the relevant search terms for “KT” and “competencies” as found mostly in titles and abstracts of the revealed publications may result in missing publications related to KT competencies. Finally, this scoping review will neither include an assessment of the identified core KT competencies, nor a critically appraisal of the effectiveness of the identified interventions/strategies focused on the development/improvement of the core competencies.

### Implications and recommendations

This scoping review will advance the KT field by incorporating the identified core KT competencies into the practice of each individual healthcare professional. The implementation of the KT competencies in practice could have several positive implications and outcomes to practice itself, education, policy, knowledge brokering, and future research. Often, knowledge synthesis studies provide recommendations based on the findings for practice. We will not be able to develop any recommendations, because the included publications will not be critically appraised for methodological quality. However, we will provide recommendations for future research on explicitly determining KT competencies for each of the three stakeholder groups (i.e., knowledge users, brokers, producers) in universities, health organizations, and decision-making centers. Further recommendations will include systematic reviews of the literature on KT competencies where sufficient knowledge exists and primary studies for areas that have not been explored yet. The findings of this scoping review could be used to guide the education of healthcare professionals and inform policy-making process. Using this scoping review as a KT tool, we will focus on the multiple domains of the identified KT competencies to capture most of the aspects and perspectives of these KT competencies for the three groups of audiences: knowledge users, knowledge brokers, and knowledge producers.

## Additional files

Additional file 1: Definitions of the primary KT concepts. A list of the primary KT concepts are described/defined for the reader convenience. (PDF 165 kb)

Additional file 2: Literature search strategy. Examples of the literature search strategies are described to be used with electronic databases and grey literature including search terms. (PDF 146 kb)

Additional file 3: Data extraction form. A description of the data extraction form is included that we will use during the data extraction phase of the proposed scoping review. (PDF 149 kb)

Additional file 4: Advisory Group. A list of the manes of Advisory Group are mentioned in alphabetical order. (PDF 143 kb)

## Abbreviations

AHRQ: Agency for healthcare research & quality
EBP: Evidence-based practice
EVIPNet: Evidence-informed policy network
EIP: Evidence-informed practice
EPCs: Evidence-based practice centers
CINAHL: Cumulative index to nursing and allied health literature
InspireNet: INnovative health Services & Practice Informed by Research & Evaluation Network
WHO: World health organization
