# Supplementary material for: Protocol of a scoping review on knowledge translation competencies
Source: Syst Rev. 2017 May 2;6:93. doi: 10.1186/s13643-017-0481-z (PMC5414292; doi:10.1186/s13643-017-0481-z)
Supplement: Supplementary file 1 — Definitions of the primary KT concepts. A list of the primary KT concepts are described/defined for the reader convenience. (PDF 165 kb) [file 13643_2017_481_MOESM1_ESM.pdf]

Appendix 1 – Definitions of the Primary KT Concepts (in alphabetical order)

Evidence & Evidence-Based Practice (medicine)

Evidence and evidence-based practice (medicine), according to the now famous definition by Sackett and colleagues (1996, p. 1) is "the conscientious, explicit, and judicious use of current best evidence in making decisions about the care of individual patients". Evidence-based practice means integration of individual professional clinical expertise, through clinical experience and practice, with the best available clinically-relevant evidence (i.e., research findings) used in the context of practitioner and patient preferences (Sackett, 1997) for patient-centered practice of the individual, population, or system (Newhouse, 2007).

Knowledge

Knowledge has been categorized as propositional (i.e., formal, explicit, derived from research that can be generalizable) and non-propositional (i.e., informal, implicit, derived primarily through practice such as tacit knowledge of professionals or personal knowledge from experience, cognitive resources, and beliefs) (Eraut 1985, 2000 as found in (Rycroft-Malone, Seers, Titchen, & Harvey, 2004). Knowledge in health is generated from different types of evidence: research, clinical experience and expertise, patient experience and preferences, and local context and environment (Rycroft-Malone et al., 2004). By combining both propositional and non-propositional knowledge we bring together the external (scientific) and internal (intuitive) approaches to knowledge.

Knowledge Brokering

Knowledge brokering is the act of bringing people together or people to information for mutual advantage in order to share learning, understand professional cultures, influence each other's work, and establish new partnerships (Canadian Health Services Research Foundation, 2003; Harris & Lusk, 2010). Knowledge brokers assist with negotiations, facilitate communication, and exchange knowledge among networks to bridge the "know-do" gap and promote evidence-based practice and evidence-informed decision-making (Lomas, 2007; van Kammen, de Savigny, & Sewankambo, 2006). For a detailed description of the suggested KB role and responsibilities, please read Glegg and Hoens (2016) work.

Knowledge Translation

Knowledge translation (KT) has been described using various terms such as applied health research, dissemination, linkage and exchange, implementation research (Graham et al., 2006), knowledge or research utilization, research uptake, knowledge mobilization, and research to action (McKibbon et al., 2010; Scott et al., 2010; Straus, Tetroe, & Graham, 2009a). More than 16 terms were identified in a theory analysis of theoretical underpinnings of KT (Graham, Tetroe, & the KT Theories Research Group, 2007, p. 939). In this proposed study, we adopted the Canadian Institutes for Health Research (CIHR) definition of knowledge translation: "a dynamic and iterative process that includes synthesis, dissemination, exchange and ethically-sound application of knowledge to improve the health of Canadians, provide more effective health services and products and strengthen the health care system" (http://www.cihr-irsc.gc.ca/e/29418.html#2). The described process takes place within a complex system of interactions between researchers and knowledge users which may vary in intensity, complexity

and level of engagement depending on the nature of research and the findings as well as the needs of the particular knowledge user. Four elements of KT are emphasized in this definition: synthesis, dissemination, exchange, and application of knowledge (http://www.cihr-irsc.gc.ca/e/29418.html#4.1). According to the CIHR website on KT, *Synthesis* means the reproducible and transparent method of contextualization and integration of research findings of primary research studies on a topic within the larger body of knowledge (e.g., systematic review). *Dissemination* activities involve identifying the appropriate audience, tailoring the message and medium to that audience, and engaging knowledge users in developing and executing an implementation plan (e.g., summaries for or briefings to stakeholders; educational sessions with patients, practitioners, and/or policy makers). *Exchange* of knowledge refers to the interaction between knowledge users and researchers that may result in mutual learning. According to the Canadian Foundation for Healthcare Improvement, knowledge exchange is defined as "collaborative problem-solving between researchers and decision-makers that happens through linkage and transfer. Effective knowledge exchange involves interaction between decision-makers and researchers and results in mutual learning through the process of planning, producing, disseminating, and applying existing or new research in decision-making." (http://tinyurl.com/z8fe65p). *Application* of knowledge is the iterative process by which knowledge is used into practice, based on ethically-sound KT activities, to improve outcomes. These KT activities are consistent with ethical principles and norms, social values, and legal regulatory frameworks (http://www.cihr-irsc.gc.ca/e/29418.html#4.1).

Knowledge Users

Knowledge user is defined by CIHR as "an individual who is likely to be able to use the knowledge generated through research to make informed decisions about health policies, programs and/or practices… A knowledge-user can be, but is not limited to, a practitioner, policy-maker, educator, decision-maker, health care administrator, community leader, or an individual in a health charity, patient group, private sector organization, or media outlet" (http://www.cihr-irsc.gc.ca/e/39033.html). For the purposes of this scoping review, knowledge users include both regulated and unregulated care providers (e.g., front-line staff working with patients/public). While we recognize there are numerous other audiences that use health research evidence (e.g., patients, public, media), we do not have the capacity to target all of them in our work. We do envision that some of these excluded individuals will have the skills and knowledge to use the KT competency pathways to be developed in the next phase of this project, when the scoping review is completed.

KT Competencies

In this scoping review, KT competencies are described as knowledge, skills, and attitudes. Our intention is to emphasize KT competencies in terms of the four elements of the KT definition (as described above): *synthesis*, *dissemination*, *exchange* and *application* of knowledge during the dynamic and iterative process of *interactions* between knowledge users, knowledge brokers and knowledge producers/researchers. Other relevant competencies that may be revealed in the literature will also include in the findings of this study.

According to the four KT elements,

1. Synthesis refers to the person (i.e., knowledge user, broker, producer/researcher) competencies as being able to search the literature including peer-reviewed publications and grey literature; understand the basics of the research process; appraise the most relevant literature/evidence/knowledge; and synthesize the available literature/evidence/knowledge (e.g., in this scoping review, we expect a number of KT competencies to be exercised as part of the KT activities).
2. Dissemination refers to distributing, for example, summaries of research findings in plain-language for stakeholders, tailored reports for knowledge users, follow-up with research participants, interactive meetings among research team members and stakeholders, summary briefings of relevant policies, videos, websites, decision aids, or art pieces. In addition, *diffusion* of newly developed knowledge (e.g., presentations in scientific conferences, publications in peer-reviewed open-access and/or traditional journals, web-based activities such as posting study findings on the web, workshops and presentations of the findings in participants and stakeholders, non-peer-reviewed publications such as newspapers and newsletters in relevant consumer advisory groups) refers to the person (i.e., knowledge user, broker, producer/researcher) competencies as being able to identify knowledge needs and preferences of target audiences; tailor key messages to target audiences; develop and implement a KT plan; understand the KT models, strategies, and activities; and evaluate a KT implementation plan.
3. Exchange refers to the person competencies to handle knowledge among interested stakeholders and being able to interpret/translate and mobilize research findings, facilitate the process of trading off knowledge, and value evidence-informed decision-making, stakeholder input and co-creation or adaptation of knowledge to the context.
4. Application of knowledge refers to the person competencies to implement knowledge into routine practices for EBP or to inform policy or research and being able to use change theories and/or models; understand research ethics and ethically-sound processes toward application of knowledge into practice or to inform policy or further research; be aware of and apply best practices in knowledge translation; understand the practice process, barriers and facilitators of evidence use; access and apply resources to carry out change; and find, understand, adapt, and implement health research knowledge (i.e., research findings) taking into account local user knowledge and context to address health/ health system issues.

Other competencies relevant to KT process may include fostering collaboration, trust, build consensus and networks, connect with people; knowing the local context and identify barriers and facilitators to target KT; and engaging with the community and diverse stakeholders including researchers and knowledge users throughout the research cycle. Depending on where in the research cycle a person is involved, there are different KT skills needed to address integrated, end-of-grant, or implementation. It also depends on whether the individual is a researcher or a knowledge user or knowledge broker.

References

Canadian Health Services Research Foundation, C. (2003). *The theory and practice of knowledge brokering in Canada's health System*.

Glegg, S. M., & Hoens, A. (2016). Role domains of Knowledge Brokering: A model for the health care setting. *Journal of Neurologic Physical Therapy, 40*(2), 115-123. doi:10.1097/npt.0000000000000122

Graham, Logan, J., Harrison, M., Straus, S., Tetroe, J., Caswell, W., & Robinson, N. (2006). Lost in knowledge translation: Time for a map? *Journal of Continuing Education in the Health Professions, 26*(1), 13-24.

Graham, I. D., Tetroe, J., & the KT Theories Research Group. (2007). Some theoretical underpinnings of knowledge translation. *Academic Emergency Medicine: Official journal of the Society for Academic Emergency Medicine, 14*(11), 936-941. doi:10.1111/j.1553-2712.2007.tb02369.x

Harris, M., & Lusk, E. (2010). *Knowledge brokering in the Canadian mental health and dementia health care system (info sheet)*.

Lomas, J. (2007). The in-between world of knowledge brokering. *BMJ : British Medical Journal, 334*(7585), 129-132. doi:10.1136/bmj.39038.593380.AE

McKibbon, K. A., Lokker, C., Wilczynski, N., Ciliska, D., Dobbins, M., Davis, D., . . . Straus, S. (2010). A cross-sectional study of the number and frequency of terms used to refer to knowledge translation in a body of health literature in 2006: a Tower of Babel? *Implementation Science, 5*(1), 16.

Newhouse, R. P. (2007). Creating infrastructure supportive of evidence-based nursing practice: Leadership strategies. *Worldviews on Evidence-Based Nursing, 4*(1), 21-29. doi:10.1111/j.1741-6787.2007.00075.x

Rycroft-Malone, J., Seers, K., Titchen, A., & Harvey, G. (2004). What counts as evidence in evidence-based practice? *Journal of Advanced Nursing, 47*(1), 81-90. doi:10.1111/j.1365-2648.2004.03068.x

Sackett, D. L., Richardson, W.S., Rosenberg, W., & Haynes, R.B. (1997).. Edinburgh, UK: Churchill Livingstone. (1997). *Evidence based medicine. How to practice and teach EBM*. Edinburgh, UK: Churchill Livingstone.

Sackett, D. L., Rosenberg, W., Gray, J. A., Haynes, R. B., & Richardson, W. (1996). Editorial: Evidence based medicine: What it is and what it isn't. *British Medical Journal (BMJ), 312*(7023), 71-72. doi:10.1136/bmj.312.7023.71

Scott, S. D., Profetto-McGrath, J., Estabrooks, C. A., Winther, C., Wallin, L., & Lavis, J. N. (2010). Mapping the knowledge utilization field in nursing from 1945 to 2004: a bibliometric analysis. *Worldviews on Evidence-Based Nursing, 7*(4), 226-237. doi:10.1111/j.1741-6787.2010.00197.x

Straus, S. E., Tetroe, J., & Graham, I. (2009a). Defining knowledge translation. *CMAJ: Canadian Medical Association Journal, 181*(3-4), 165-168. doi:10.1503/cmaj.081229

van Kammen, J., de Savigny, D., & Sewankambo, N. (2006). Using knowledge brokering to promote evidence-based policy-making: The need for support structures. *Bulletin of the World Health Organization, 84*(8), 608-612.
