# Supplementary material for: Protocol of a scoping review on knowledge translation competencies
Source: Syst Rev. 2017 May 2;6:93. doi: 10.1186/s13643-017-0481-z (PMC5414292; doi:10.1186/s13643-017-0481-z)
Supplement: Supplementary file 2 — Literature search strategy. Examples of the literature search strategies are described to be used with electronic databases and grey literature including search terms. (PDF 146 kb) [file 13643_2017_481_MOESM2_ESM.pdf]

Appendix 2 – Literature Search Strategy

<u>Electronic online databases to be searched</u>

1. PubMed / MEDLINE
2. Cumulative Index to Nursing and Allied Health Literature (CINAHL EBSCO)
3. Cochrane Library (Wiley)
4. EMBASE (Ovid)
5. NEOS Library Consortium Catalogue
6. Scopus
7. Theses Canada

<u>Grey literature sources to be searched</u>

1. Evidence-Informed Healthcare Renewal (EIHR) Portal
2. INnovative health Services and Practice Informed by Research & Evaluation NETwork (InspireNet) Grey Literature database
3. KT Clearinghouse
4. Center on Knowledge Translation for Disability and Rehabilitation Research (KTDRR) KT strategies' database
5. Quebec Population Health Research Network (formerly KUUC)
6. Google (and other KT websites as discovered)
7. UK National Institute for Health and Care Excellence (NICE)
8. World Health Organization (WHO)
9. Working group and stakeholders input

<u>Search strategies</u>

The conceptual building blocks for the search are outlined below. The actual search terms entered into databases and search engines will include their variants (e.g. singular, plural, British / American spelling, truncation, etc.) and be selected according to the characteristics and functionalities of the specific database.

Search terms

KT Keywords (combined with OR, searched in Title field)	AN D	Competencies Keywords (combined with OR)
• knowledge transfer<br>• knowledge utilization<br>• knowledge use<br>• knowledge translation<br>• knowledge implementation<br>• research in practice<br>• knowledge mobilization<br>• knowledge exchange<br>• research transfer<br>• research utilization<br>• research use<br>• research dissemination<br>• knowledge dissemination<br>• research exchange<br>• research translation<br>• knowledge to action<br>• know do gap<br>• evidence informed<br>• diffusion of knowledge<br>• research into practice<br>• knowledge into practice<br>• evidence into practice<br>• translational science		• Competenc*<br>• Capacity building<br>• Skill*<br>• Ability<br>• Training<br>• Curriculum<br>• Learning

*indicates truncation
