# Supplementary material for: Protocol of a scoping review on knowledge translation competencies
Source: Syst Rev. 2017 May 2;6:93. doi: 10.1186/s13643-017-0481-z (PMC5414292; doi:10.1186/s13643-017-0481-z)
Supplement: Supplementary file 3 — Data extraction form. A description of the data extraction form is included that we will use during the data extraction phase of the proposed scoping review. (PDF 149 kb) [file 13643_2017_481_MOESM3_ESM.pdf]

Appendix 3 – Data Extraction Form

Reviewer:	
Date:	
**Publication Information**	
Study unique ID:	
1st Author's Name:	
Publication Year:	
Journal:	
Country:	
**Demographics**	
Population/Sample:	
Study Design:	
**Theoretical Framework**	
Yes (specify):	No
**KT competencies**	
Knowledge (specify):	• • •
Skills (specify):	• • •
Attitudes (specify):	• • •
**Data analysis**	
Quantitative:	
Qualitative:	
Not applicable:	
**Study Results**	
Main findings:	• • •
**Interventions/Strategies**	
Yes (specify):	No
What was the focus of the intervention?	
Description of the intervention:	
**Conclusion**	
Include:	
Exclude (provide rationale):	
Additional Comments:	
