# Supplementary material for: Protocol of a scoping review on knowledge translation competencies
Source: Syst Rev. 2017 May 2;6:93. doi: 10.1186/s13643-017-0481-z (PMC5414292; doi:10.1186/s13643-017-0481-z)
Supplement: Supplementary file 4 — Advisory Group. A list of the manes of Advisory Group are mentioned in alphabetical order. (PDF 143 kb) [file 13643_2017_481_MOESM4_ESM.pdf]

Appendix 4 – Advisory Group

(in alphabetical order)

- **Donna Angus**, Knowledge Translation Consultant, Edmonton, Alberta, Canada.
- **Moriah Ellen**, Investigator, McMaster Health Forum's Impact Lab; Associate Professor, Jerusalem College of Technology; Senior Researcher, Gertner National Institute for Health Policy, Hamilton, Ontario, Canada.
- **Alison Hoens**, Physical Therapy Knowledge Broker, Department of Physical Therapy, Faculty of Medicine, University of British Columbia; Research, Education and Practice Coordinator for Physiotherapy, Providence Health Care, Vancouver, British Columbia, Canada.
- **Bev Holmes**, Vice-President, Research & Impact, Michael Smith Foundation for Health Research, Vancouver, British Columbia, Canada.
- **Sheila Kerr**, Member, Arthritis Patient Advisory Board, Arthritis Research Canada, Vancouver, British Columbia, Canada.
- **Linda Li**, Senior Research Scientist of Clinical Epidemiology, Arthritis Research Canada; Harold Robinson/Arthritis Society Chair in Arthritic Diseases; Canada Research Chair in Patient-Oriented Knowledge Translation; Associate Professor, Department of Physical Therapy, University of British Columbia, Vancouver, British Columbia, Canada.
- **Grace Mickelson**, Corporate Director - Academic Development, Provincial Health Services Authority (2005 - 2015), Vancouver, British Columbia, Canada.
- **Kelly Mrklas**, Knowledge Translation Implementation Scientist; Director, Knowledge for Change Unit, Alberta Health Services, Calgary, Alberta, Canada.
- **Laura Mumme**, Knowledge Mobilization Consultant, Alberta Health Services, Edmonton, Alberta, Canada.
- **Katrina Plamondon**, Regional Practice Leader, Research & Knowledge Translation, Interior Health, Kelowna, British Columbia, Canada.
- **Anne Sales**, Associate Chair for Education Programs and Health System Innovation; Professor, Division of Learning and Knowledge Systems; Professor of Nursing, Department of Systems, Populations and Leadership; Director, Health Infrastructures and Learning Systems (HILS) MS and PhD Program, School of Medicine, University of Michigan; Research Scientist, Centre for Clinical Management Research, VA Ann Arbor Healthcare System, Michigan, USA.
- **Victoria Schuckel**, Acting Executive Director, Research, Knowledge Translation & Library Services, British Columbia Ministry of Health, Victoria, British Columbia, Canada.
- **Denise Thomson**, Associate Director, Knowledge Translation Platform, Alberta SPOR SUPPORT Unit, Edmonton, Alberta, Canada.
